# Ecological immunology in a fluctuating environment: an integrative analysis of tree swallow nestling immune defense

**DOI:** 10.1002/ece3.504

**Published:** 2013-03-09

**Authors:** Gabriel Pigeon, Marc Bélisle, Dany Garant, Alan A Cohen, Fanie Pelletier

**Affiliations:** 1 Département de biologie, Université de Sherbrooke2500 boulevard de l'Université, Sherbrooke, QC, J1K 2R1, Canada; 2 Canada Research Chair in Evolutionary Demography and Conservation, Département de biologie, Université de Sherbrooke2500 boulevard de l'Université, Sherbrooke, QC, J1K 2R1, Canada; 3 Département de médecine de famille, Université de Sherbrooke3001 12è Ave Nord, Sherbrooke, QC, J1H 5N4, Canada

**Keywords:** Agricultural intensification, bird, ecological immunology, integrated immune score, performance, tree swallow

## Abstract

Evolutionary ecologists have long been interested by the link between different immune defenses and fitness. Given the importance of a proper immune defense for survival, it is important to understand how its numerous components are affected by environmental heterogeneity. Previous studies targeting this question have rarely considered more than two immune markers. In this study, we measured seven immune markers (response to phytohemagglutinin (PHA), hemolysis capacity, hemagglutination capacity, plasma bactericidal capacity, percentage of lymphocytes, percentage of heterophils, and percentage of eosinophils) in tree swallow (*Tachycineta bicolor*) nestlings raised in two types of agro-ecosystems of contrasted quality and over 2 years. First, we assessed the effect of environmental heterogeneity (spatial and temporal) on the strength and direction of correlations between immune measures. Second, we investigated the effect of an immune score integrating information from several immune markers on individual performance (including growth, mass at fledging and parasite burden). Both a multivariate and a pair-wise approach showed variation in relationships between immune measures across years and habitats. We also found a weak association between the integrated score of nestling immune function and individual performance, but only under certain environmental conditions. We conclude that the ecological context can strongly affect the interpretation of immune defenses in the wild. Given that spatiotemporal variations are likely to affect individual immune defenses, great caution should be used when generalizing conclusions to other study systems.

## Introduction

Wild organisms are continuously exposed to pathogens such as viruses, bacteria, or macroparasites. An individual's immune capacity is thus closely linked to its fitness (Saino et al. 2004; Cichon and Dubiec 2005). Several studies have shown that environmental conditions can affect an organism's immune defenses (Neve et al. 2007; Arriero 2009). Understanding why and how biotic and abiotic factors contribute to variation in immunity in free-living organisms is therefore critical to understanding the evolution of physiological systems (Martin et al. 2011). The fact that environmental conditions can interact with an organism's immune defenses has prompted the emergence of a new field often referred to as ecological immunology. Given that immune defenses are costly to develop, maintain, and use (reviewed in Lochmiller and Deerenberg 2000), individuals are expected to trade-off limited resources between immunity and other costly functions such as growth and reproduction. Maximization of an organism's immune defenses is also limited due to the risk of an over-responsive immune system, which can lead to autoimmune pathology (Graham et al. 2005; Sadd and Siva-Jothy 2006). Immune defenses are thus expected to be under stabilizing selection. For example, in blue tits (*Cyanistes caeruleus*), individuals at both extremes of antibody responsiveness were shown to have lower survival probability (Raberg and Stjernman 2003). The optimal level of immune defense, however, is not fixed because different environments have different pathogen pressures, which can lead to local optima (Viney et al. 2005). Relating immunity and fitness is therefore not a trivial task because the environment and immune system are involved in complex relationships.

An organism's immune defenses are the result of a complex network system composed of many effectors or functions that deal with infections originating from diverse pathogens (Segel and Cohen 2001). This complexity causes important challenges for researchers interested in studying the immune system in an ecological context. Until recently, few studies had simultaneously considered more than two immune indices. To bring new insights to our understanding of eco-immunology, researchers have come to the consensus that multiple immune indices should be used to quantify the immune defenses of an individual. For example, Buehler et al. (2011) found that several immune indices of red knots (*Calidris canutus*) varied significantly over the annual cycle. Similarly, immune functions of house sparrows (*Passer domesticus*) have been found to change during the annual cycle (Pap et al. 2010). Immune functions can also change according to environmental constraints as red knots in captivity appear to use different immune strategies than wild individuals (Buehler et al. 2008). Correlations between immune measures can also vary. Matson et al. (2006a) measured 13 immune markers and detected positive correlations between functionally related immune markers, such as hemolysis and hemagglutination, within waterfowl (Anseriformes) species. However, those correlations were no longer significant when compared across species, suggesting different interspecific immune constraints. Correlations among immune markers could also vary within a species. In the wild, the correlation between phytohemagglutinin (PHA)-induced inflammation and humoral immune response differs between tree swallows (*Tachycineta bicolor*) living in Tennessee and those living in New York or Alaska (Ardia 2007). The rearing environment is also known to modify the correlation between PHA response and immunoglobulin level in blue tit nestlings (Arriero 2009). Although few studies have investigated within-population differences, based on patterns observed across populations, different environmental conditions within populations are also expected to affect the strength and direction of correlations among immune indices. For example, low-quality habitat could generate trade-offs between different components of the immune defenses leading to negative correlations.

The aim of this study was to assess the effects of environmental quality and annual variation on the correlations among seven immune markers in tree swallow nestlings raised in contrasted agro-ecosystems of southern Québec, Canada. Previous studies on this system have revealed large differences in environmental quality both over time and space (Ghilain and Bélisle 2008; Rioux Paquette et al. In press; Baeta et al. 2012; see study area section). More specifically, we assessed: (1) if correlations between different immune measures changed across habitat and year in a predictable manner, and (2) if an integrated immune score correlated with individual performance. We used seven immune measures to assess the immune system of nestlings. Those measures included (1) the response to phytohemagglutinin (PHA), a measure of induced pro-inflammatory capacity (Vinkler et al. 2010); (2) the hemolysis capacity, a measure of natural antibody levels; (3) the hemagglutination capacity, a measure of the complement activation (Matson et al. 2005); (4) the bactericidal capacity of plasma, a measure of the capacity to eliminate bacterial pathogens (Matson et al. 2006b); (5) the percentage of lymphocytes, a measure of immunological investment (Beldomenico et al. 2008); (6) the percentage of heterophils, non-specific, phagocytizing cells that are modulators of inflammatory responses (Maxwell and Robertson 1998); and (7) the percentage of eosinophils, which play a role in inflammation and defense against parasites (Davis et al. 2008). We then combined a multivariate and a simpler pair-wise correlation approach (as suggested in Buehler et al. 2011) to assess how environmental heterogeneity affects the consistency of relationships among immune measures and to evaluate the effect of immunity on three proxies of individual performance (fledgling mass, nestling growth, and parasite burden).

## Materials and Methods

### Study area and population

**Figure 1:**
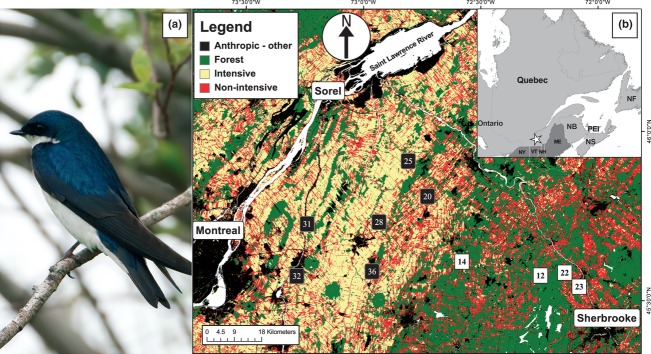
A) Adult tree swallow (*Tachycineta bicolor*) on a branch and B) distribution of the 10 farms used in this study and located along a gradient of agricultural intensification in southern Québec, Canada, 2010–2011. The location of the study area at larger scale is represented by a star on the map in the top right corner. Land cover types (represented by the colours black, green, yellow and red) are based on a mosaic of classified LANDSAT-7 satellite images (Canadian Wildlife Service 2004). Dark squares indicate intensive farm locations and white squares indicate extensive farm locations. Photo by Gabriel Pigeon.

Tree swallows are small aerial insectivores (mean ± SD = 21.9 ± 1.5 g) that breed during summer over most of North America. They are mostly found in open habitats near water or agricultural fields (Winkler et al. 2011). Our study was conducted using nest-boxes established in 2004 in southern Québec, Canada, over a 10,200 km^2^ area (Fig. 1). Although the breeding activities of tree swallows have been followed yearly since 2004, monitoring of immune measures started in 2010. Therefore, this study focuses on tree swallow nestlings born in 2010 and 2011 on 10 farms (10 nest-boxes/farm) located at both extremes of a gradient of agricultural intensification (i.e., 4 farms in extensively cultivated farmlands and 6 farms in intensively cultivated farmlands). Agricultural intensification is characterized by a shift to higher productivity cultivars grown as part of large monocultures, a reduction in marginal habitats and increased use of pesticides and fertilizers (Donald et al. 2006). It has been associated with a decline of farmland bird populations in many countries (Chamberlain et al. 2000; Murphy 2003) and to a greater extent, of aerial insectivorous birds such as tree swallows (Blancher et al. 2009; Nebel et al. 2010). The mechanisms through which agricultural intensification negatively affects bird populations involve the homogenization of habitat and landscape structure as well as pesticide use which are all hypothesized to lower insect prey abundance (Benton et al. 2002). For instance, insect prey availability within intensively cultivated landscapes has been shown to be lower than in extensively cultivated ones in our study system (Rioux Paquette et al. In press). Moreover, the clutch size and fledging probability of tree swallows decrease with increasing amounts of intensively cultivated areas within 5 km of our nest-boxes (Ghilain and Bélisle 2008). Nest-box occupancy by tree swallows within intensive farmlands of our study system is also lower due to competition with house sparrows (Robillard et al. 2012). All of these evidences support the assumption that extensively cultivated landscapes consist in higher quality habitats compared to intensively cultivated ones.

We accordingly assigned nest-boxes to either intensive (low-quality habitat) or extensive (higher quality habitat) agricultural landscapes on the basis of the relative cover of intensive and extensive cultivars found within a radius of 500 m and 5 km of nest-boxes. We defined intensive agricultural landscapes as those mostly composed of annual crops, such as corn, soybean, and other cereals, and extensive ones as those mostly composed of hayfields, pastures and fallows (for more details on landscape characterization see Ghilain and Bélisle 2008). At the 500-m scale, cultivars were identified visually in the field on each year and reported on orthophotos (1:40 000). The relative cover of intensive and extensive cultivars was then calculated using ArcView GIS Spatial Analyst 2.0a (ESRI 2005). At the 5-km scale, the relative cover of intensive and extensive cultivars was also measured with ArcView, but based on a mosaic of geo-referenced and classified Landsat-7 satellite images taken between August 1999 and May 2003 (pixel resolution 25 × 25 m; Canadian Wildlife Service 2004). The relative cover at both the 500-m and the 5-km scale was highly correlated (intensive-2010: *r* = 0.90; intensive-2011: *r* = 0.84; extensive-2010: *r* = 0.75; extensive-2011: *r* = 0.67), leading to the same classification of nest-boxes over both years.

### Blood sampling

We collected blood samples of nestlings on day 8 after hatching from the left brachial vein using heparinized capillaries. The first 5 μL was used for leukocyte counts. An additional 30 μL was dried on filter paper to determine sex based on DNA (for details, see Porlier et al. 2009). All remaining blood was immediately put on ice and centrifuged within 45 min. Capillaries were centrifuged 10 min at 14,500 g (using a Clinical 200 centrifuge, VWR, Quebec, Canada) before freezing. Plasma was kept frozen until used for agglutination and bacteria killing assays (see below). If the total plasma volume collected was inferior to 50 μL, nestlings were re-sampled 2 days later and both plasma samples homogenized to obtain a sufficient volume for assays (76% of 210 nestlings were re-sampled). Differing proportion of plasma of 8- versus 10-day-old nestling had no significant effect on relevant immune (linear mixed model; bactericidal capacity: coef. = 0.01 ± 0.06 SE, *P* = 0.82; hemagglutination: −0.10 ± 0.12, *P* = 0.40; and lysis: −0.32 ± 0.33, *P* = 0.34). This is similar to results from Palacios et al. (2009), who found no significant effect of nestling age on lysis or hemagglutination.

### Immunity measurements

#### PHA response

We measured pro-inflammatory response by measuring the response to phytohemagglutinin (PHA; Martin et al. 2006; Pigeon et al. In press). Nestlings were tested on day 14 after hatching. Each bird was injected 0.1 mL of PHA 1 mg/mL (L8754-50MG, Sigma Aldrich, St Louis, MO, USA) diluted in phosphate-buffered saline (no. 811-010-CL, Wisent, St-Bruno, Canada) in the left wing patagium. PHA response was measured as the difference in patagium thickness (in mm) before and after PHA injection as determined with a screw micrometer (293 MDC-Lite, Mitutoyo Corporation, Montreal, Canada, ±0.001 mm). The second patagium measurements were taken 48 h following injection (mean ± SD = 47.92 ± 0.49 h). All patagium measurements were made by the same observer. Repeatability of PHA measurements, defined as the proportion of the total variation that can be attributed to variation among individuals versus variation among measurements within individuals (Wolak et al. 2012), was very high *r* = 0.97, *n* = 408 nestlings, 3 repeated.

#### Agglutination and lysis

We measured natural antibodies and complement effectiveness using a hemolysis-hemagglutination assay following the protocol of Matson et al. (2005) modified by Palacios et al. (2009). Briefly, 10 μL of plasma was used for a serial dilution (1:2) in phosphate-buffered saline in a 96-well u-bottom plate (3797, Corning Inc., Corning, NY, USA) to obtain 11 plasma concentrations with a negative control for each sample. We then added 10 μL of a 2% rabbit red blood cell solution (R309-0050, Rockland Immunochemicals, Gilbertsville, PA) to each sample and incubated them 90 min at 37ºC, then 20 min at 20ºC inclined at 45º before scanning the samples at 300 dpi resolution (HP CM1312nfi mfp). Agglutination and lysis were scored from the scan from 1 to 12 (the negative log2 of the last plasma dilution exhibiting agglutination and lysis as in Matson et al. (2005)). We tested individuals in duplicate when sufficient plasma was available (*n* = 175 of 210) and used mean score in statistical analyses. All scoring was done by G. Pigeon blind to nest-box location. To avoid storage effects on measurements, scoring was performed within 2 months after the field season. Therefore, the year was known to the observer, but the average scoring in respective years was unknown.

#### Bacteria killing assay

We measured the bactericidal capacity of plasma following the bacteria killing assay protocol used by Morrison et al. (2009) (modified from Matson et al. 2006b). *E. coli* pellets (ATCC 8739) were reconstituted and diluted in PBS to obtain a concentration of 100–150 CFU per plate on controls. We then added 5 μL of plasma to 20 μL of this bacteria solution and 95 μL of cell culture medium. This solution was incubated 45 min at 40ºC before plating 50 μL on LB plates. A negative control without tree swallow plasma was made every hour. All plates were performed in duplicate and incubated for 24 h at 40ºC. The bactericidal capacity of plasma was recorded as [1−(average number of surviving colonies on an individual's plates/average number of colonies on control plates)] × 100.

#### White cell counts

To obtain a leukocyte profile, we smeared and air dried approximately 5 μL of fresh blood on a glass slide immediately after collection on 8-day-old nestlings. Smears were stained with quick-dip stain and counter stain without heat fixation (Toma et al. 2006) before they were scanned for leukocytes using a 10× ocular and 100× oil immersion lens. A total of 100 leukocytes were identified from each slide, noting the frequencies of lymphocytes, heterophils, eosinophils, basophils, and monocytes. Leukocytes were identified according to Clark et al. (2009). Basophils and monocytes were not considered further due to their rarity (<1%). A sub-sample of nine slides was counted four times to estimate repeatability (Wolak et al. 2012). Repeatability (*r*) was 0.75, 0.91, and 0.63 for lymphocytes, heterophils, and eosinophils, respectively.

### Proxies of individual performance

We considered three proxies of individual performance: growth between 2 and 16 days after hatching, mass at fledging and parasite burden. Nests were monitored every 2 days during the breeding season (from early May to mid-July). Nestlings were marked individually by nail clipping until their 12th day of life and thereafter ringed with an official aluminum ring. Nestlings were weighed (± 0.01 g) with a platform scale equipped with a contention device at 2 and 16 days after hatching. Growth was calculated as the mass gained between those two measurements. Mass at day 16 was used as fledging mass. The number of *Protocalliphora* pupae in each nest was counted after the breeding season and used as a measure of parasite burden imposed by a blood-feeding ectoparasite (Daoust et al. 2012). These three measures of performance influence survival and were used as we could not obtain more direct measures of fitness. Growth and fledging mass are positively correlated with the probability of recruitment in several species of birds, including tree swallow (McCarty 2001; Monros et al. 2002), whereas a large ectoparasite burden significantly reduces survival (Thomas et al. 2007). All procedures described in this study were approved by the Université de Sherbrooke's Animal Care Committee (protocol number FP2009-01) and comply with current Canadian laws regarding animal research.

### Statistical analysis

#### Assessment of the data structure

We only retained nestlings for which all immunity measures were available for analyses (*n* = 210). We checked all immune measures for normality. Bactericidal capacity of plasma was highly skewed; we therefore normalized it prior to all analyses using the following formula: -√(1-bactericidal capacity) (Legendre and Legendre 1998). Immune measures were then centered and scaled to unit variance to reduce the impact of measurement units. Given the statistically inherent correlations among the percentages of three types of leukocytes (−0.67 ≤ *r* ≤ −0.30), only one leukocyte percentage was used in multivariate analysis. We used lymphocyte percentage as an indicator of immunological investment (Beldomenico et al. 2008). We conducted a redundancy analysis (RDA), which is an extension of multiple linear regression to multivariate response variables (Legendre and Legendre 1998) on immunity measurements (bactericidal capacity, hemolysis, hemagglutination, response to PHA and percentage of lymphocyte) using year, farmland type, sex and all possible two way interactions as constraining variables to assess the general correlation structure among all five immunity variables as well as the impact of possible grouping variables on their correlation structure. The RDA was conditioned on nest-box to control for non-independence of nestlings from the same nest-box. We then used an ANOVA-like permutation test for RDA using 1000 permutation to assess the level of significance (Legendre et al. 2011). The model was then simplified in a step-wise fashion. The final model included farmland type, year and their interaction. The RDA and the permutation test were performed using the vegan v2.0-4 package in R (Oksanen et al. 2012). Given that both year (2010, 2011) and farmland type (extensive, intensive) and their interaction had significant effects (Table 2), we considered that our population consisted of four sub-groups, representing different ecological contexts (intensive-2010, intensive-2011, extensive-2010 and extensive-2011). Normalized immune measurements were centered and scaled to unit variance within each sub-group for further analyses. The above analyses were performed in R v2.15. (R Development Core Team 2012).

#### Correlation among immunity indices

We followed the statistical methods suggested by Buehler et al. (2011) for assessing the relationships among immunity indices and their variability across years and farmland types. First, principal component analysis (PCA) and correlation circles were obtained for each of the four sub-groups using the ade4 package in R (Dray 2007). We examined correlation circles for principal components (PC) with an eigenvalue higher than one (i.e., for PC 1 and 2 in all cases, as well as for PC 3 for extensive-2010) (Electronic, Table S2). To help interpreting the group-specific PCA axes, we used a pair-wise approach to evaluate the correlations among all our immune markers. We thus calculated a Pearson's correlation coefficient and its 95% confidence intervals with the Fisher Z-score method following Sokal and Rohlf (1994) for every pair of immune measures by year and by farmland type. These correlation analyses were also performed in R.

#### Correlation between immunity and proxies of individual performance

We used the scores on the first and second principal components provided by the group-specific PCAs as general immunity indices. The relationships between immunity and fledging mass and growth were modeled in R using linear mixed models (Bates et al. 2011) in order to take into account the non-independence of nestlings born in the same nest-box. None of the models in which the effect of PC1 was allowed to vary by nest-box were significantly better than when only the intercept varied (most significant: *χ*^2^ = 3.08, *P* = 0.21), so a common slope was used. As burden was assessed at the nest-box level, the relationship between immunity and parasite burden was modeled with a linear model using the average immune score by nest as response variable.

## Results

### Assessing data structure

We measured the immunity of 210 tree swallow nestlings over the 2 years of the study (Table 1). Preliminary RDAs of the entire dataset suggested a difference between years as well as between habitats of different agricultural intensity (Electronic, Fig. S1). RDA1 and RDA2 explained, respectively, 16.38% and 3.98% of the variance in the 5 immune indices (Table S1). The permutation test supported this interpretation: year, farmland type, and their interaction, but not nestling sex (Variance = 0.02, *F*-value = 1.18, *P*-value = 0.31), had significant effects on the measures of nestlings' immunity (Table 2). Hence, further analyses were performed on four sub-groups representing both farmland types and years.

**Table 1:** Descriptive statistics of seven immune measures taken on tree swallow nestlings in southern Québec, Canada, 2010 (*n* = 128) and 2011 (*n* = 82)

Immune marker	Mean	Median	Minimum	Maximum
PHA	1.10	1.11	0.02	2.26
Bacteria killing	0.72	0.85	0.00	1.00
Hemolysis	1.21	1.00	0.00	3.50
Agglutination	8.22	8.25	3.00	12.00
% lymphocytes	44.63	45.00	15.00	79.00
% heterophils	28.89	29.00	6.00	58.00
% eosinophils	25.42	25.00	6.00	51.00

**Table 2:** Effects of different grouping variables on five immune measures taken on tree swallow nestlings in southern Québec, Canada (2010–2011; see Table 1) and assessed by redundancy analysis. Heterophil and eosinophil percentages were ignored because of non-independence with lymphocyte percentage. *P*-values were obtained based on ANOVA-like permutation tests conditioned by nest-boxes using 1000 permutations. Sample size is 210

Grouping variable	Variance	*F*-value	*P*-value
Year	0.73	37.86	<0.001
Farmland type	0.08	4.27	0.001
Year: Farmland type	0.13	6.48	<0.001

### Relationships among immune measures across environments

**Figure 2:**
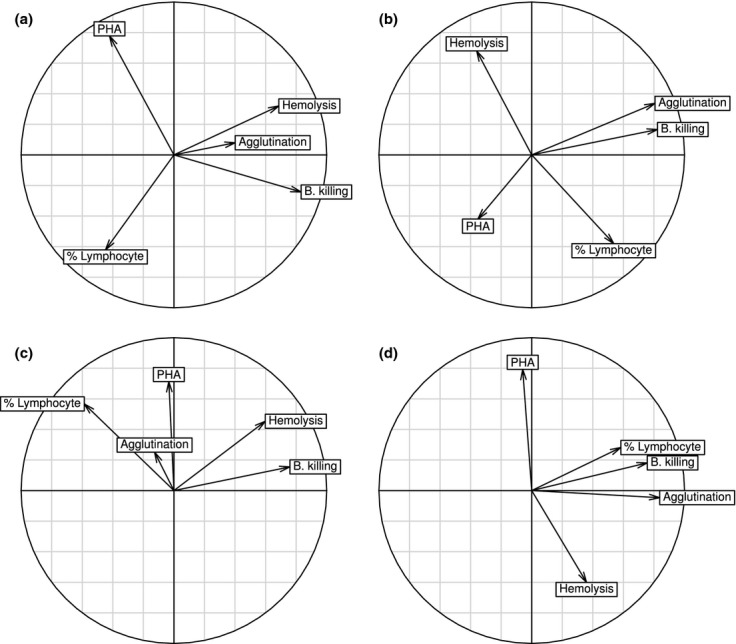
Correlation circles representing the association between immune measurements for each farmland-year sub-group of tree swallow nestlings studied in southern Québec, Canada, 2010–2011. (a) Intensive 2010: *n* = 38, 57.25% of variance explained by both PCs; (b) Intensive 2011, *n* = 27, 59.77%; (c) Extensive 2010, *n* = 90, 48.37%; (d) Extensive 2011, *n* = 55, 57.01%. Immune measures included the response to PHA (PHA), the bacteria killing capacity (B. killing), the hemolysis and hemagglutination scores and the percentage of lymphocytes. The horizontal and vertical axes represent PC1 and PC2 of a principal component analysis applied to each farmland-year sub-group. The lengths of the vectors indicate the strengths of the relationships. The size of the angle between vectors indicates the direction of the correlation: a small angle between two vectors represents a positive correlation; a 90° angle indicates no correlation and opposed vectors indicates a negative correlation.

The first two components of the sub-group PCAs explained together from 48.37% (in extensive-2010) to 59.77% (in intensive-2011) of the variance (mean = 55.60%). All four sub-groups showed different correlation patterns. Hemolysis and lymphocyte percentage showed a negative correlation (long opposed [151º and 165º] vectors) in both intensive sub-groups, in contrast to extensive sub-groups (either perpendicular (90°) vectors or long and small vectors angled at 99º and 85º; Fig. 2). Agglutination was strongly positively correlated with bacteria killing capacity in both intensive-2011 (11º between vectors) and extensive-2011 (17º between vectors), but not in extensive-2010 sub-groups (105º between vectors) or intensive-2010 (28º between vectors, and short vector length for agglutination; Fig. 2). Relationships between hemolysis and agglutination were always low. Bacteria killing capacity and percentage of lymphocytes showed no correlation except in extensive 2011 where the relationship was positive (12º between vectors; Fig. 2).

**Figure 3:**
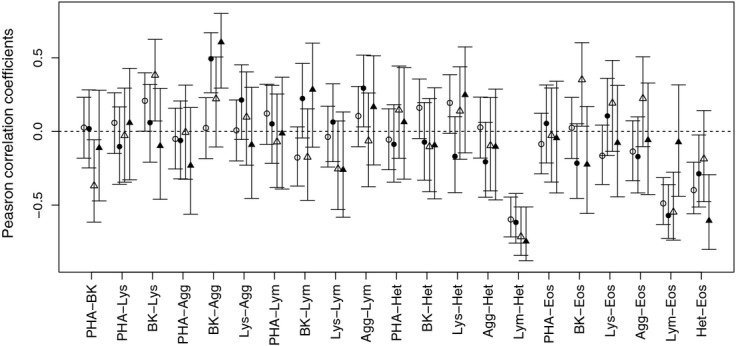
Pair-wise Pearson correlation coefficients among immune measures of nestling tree swallows studied in southern Québec, Canada, 2010–2011, according to levels of agriculture intensity (extensive = circles; intensive = triangles) and year (2010 = filled shape; 2011 = open shape). Error bars represent 95% confidence intervals calculated by Fisher Z-score transformation. All possible pairs among phytohemagglutinin response (PHA), hemagglutination (Agg), hemolysis (Lys), bacteria killing capacity of plasma (BK), percentage of lymphocytes (Lym), percentage of heterophils (Het), and percentage of eosinophils (Eos) were included.

We used a pair-wise approach to explore correlations among indices, which showed that sub-groups differed in the strength and direction of correlations among different immune markers (Fig. 3). Correlation coefficients ranged from −0.36 to 0.61. There was considerable variation even within sub-groups, resulting in large confidence intervals. Both year and farmland type affected the strength and direction of several pair-wise correlations. For instance, the positive correlation between bacteria killing capacity and agglutination titer was significant only in 2011 (intensive: *r* = 0.61, *P* < 0.001; extensive: *r* = 0.49, *P* = 0.001). Also, the correlation differed significantly between extensive-2010 (*r* = 0.02, *P* = 0.82) and extensive-2011. In contrast, the correlation between bacteria killing capacity and hemolysis was significant in 2010 (intensive: *r* = 0.38, *P* = 0.02; extensive: *r* = 0.21, *P* = 0.05), but not in 2011 (intensive: *r* = −0.10, *P* = 0.63; extensive: *r* = 0.06, *P* = 0.66). Additionally, only extensive-2011 had a significant agglutination–lymphocyte correlation (*r* = 0.29, *P* = 0.03). Although none of the four sub-group correlations between hemolysis and lymphocyte percentage differed significantly, farmland type seemed to have a greater effect on the correlations than did year. Only in intensive-2010 did a significant negative correlation between two immune indices occur, namely between PHA response and bacteria killing capacity of plasma (*r* = −0.37, *P* = 0.02). To assess whether our results were due to a lack of independence among immunity measurements of nestlings originating from the same nest, we also ran the analyses using the average immune indices from nest-boxes. These analyses (not shown) led to similar conclusions. Overall, the relationships between immune indices were inconsistent among sub-groups in accordance with the results obtained using principal component analysis.

### Linking integrated immune score and individual performance

Growth and fledging mass were highly positively correlated (*r* = 0.86, *P* < 0.001, *n* = 210), while parasite burden was moderately negatively correlated with fledging mass and growth (*r* = −0.34, *P* < 0.001, *n* = 210 and −0.37, *P* < 0.001, *n* = 210, respectively). Given that PCA was performed by sub-group, the integrated immune score implicitly differs among sub-groups. The relative importance of each immune measure on the PC1 axis is represented by the vector length on the horizontal axis of the correlation circles (Fig. 2). Mass at fledging was negatively correlated with PC1 only in the intensive-2010 sub-group (Table 3). Similarly, growth was negatively correlated with PC1 in intensive-2010; however, the effect was only marginally non-significant (Table 3). Individuals with a higher integrated immune score (mostly bacteria killing, hemolysis and agglutination) had higher mass at fledging and growth in intensive-2010 (electronic supplementary material, Fig. S2–S3). Nest-box identity explained a large portion of the variance in fledging mass (mean ± SD = 43.29 ± 11.69%) and growth (63.18 ± 6.16%). None of the sub-groups had a significant relationship between PC2 and fledging mass or growth. The linear regression using nest average immune score revealed that the relationship between immunity and parasite burden were non-significant (Table 3). However, extensive-2011 showed a marginally significant negative relationship. Averaging by nest-box greatly reduced our statistical power, especially in intensive farmlands where sample size was already small due to lower nest-box occupancy. Yet, parasite burden in extensive-2011 showed a marginally significant negative relationship with PC1, suggesting that individuals with a lower immune score had higher parasite burden (see Fig. S4).

**Table 3:** Effect of integrated immune response (PC1) on A) mass at fledging, B) growth between day 2 and 16 after hatching, and C) parasite burden (number of *Protocalliphora* pupae in nest) of tree swallow nestlings for each farmland-year sub-group (intensive 2010, intensive 2011, extensive 2010 and extensive 2011). Estimates (± standard error (SE)) were obtained for linear mixed models including nest-box as a random effect for mass at fledging and growth and from a linear model for parasites. % refers to the proportion of total variance explained by nest-box identity

Sub-group		Intensive 2010	Intensive 2011	Extensive 2010	Extensive 2011
No of nestling (no of nest)		38 (11)	27 (10)	90 (25)	55 (16)
A) Fledging mass	Estimate	0.485	−0.235	−0.019	0.234
	SE	0.221	0.273	0.173	0.198
	*F*-value	4.820	0.742	0.012	1.387
	*P*-value	0.037	0.40	0.91	0.25
	%	39.86	28.53	49.68	55.10
B) Growth	Estimate	0.034	−0.009	0.003	0.000
	SE	0.018	0.028	0.014	0.015
	*F*-value	3.444	0.101	0.034	0.001
	*P*-value	0.075	0.76	0.85	0.98
	%	71.25	56.88	60.35	64.24
C) Parasites	Estimate	−6.181	3.171	−1.967	−6.628
	SE	4.258	4.988	3.559	3.359
	*F*-value	1.452	0.636	0.553	1.973
	*P*-value	0.18	0.54	0.58	0.06

## Discussion

Our results highlight the importance of considering environmental heterogeneity when assessing individual immune defenses. Indeed, the relationships among immune measures of nestling tree swallows varied across agricultural environments. Yet, the spatial differences in environments had important effects that varied through time. Moreover, while nestlings with greater immune scores sometimes performed better, the magnitude of the effect was weak and depended on the environment. Our results reinforce the claim that the complexity and variability of the immune system may not be captured by one or two immune markers (Matson et al. 2006a; Ardia 2007). While the use of multiple immune markers is a significant step forward for ecological immunology, future studies will also need to overcome the challenges caused by temporal and spatial environmental differences.

### Relationships among immune measures across environments

To face a high diversity of pathogens, the immune system must be flexible, which might explain why correlations were not consistent between environments. Matson et al. (2006a) previously showed that relationships among immune indices changed across species and Ardia (2007) showed that within a species (tree swallows) they changed across populations. Our results now suggest that there is variation even within a single population and that correlations vary in an unpredictable manner at the relatively large spatiotemporal scales that we used (as suggested by a significant year by farmland-type interaction). Our results suggest that local environment (both spatial and temporal) can influence the correlation pattern among immune markers and hence, nestlings' immune defense.

We expected that investment trade-offs among immune functions, resulting in negative correlations between competing immune components, would be observable in intensive farmlands, which are composed of lower quality habitats (Ghilain and Bélisle 2008; Arriero 2009). The PHA response and the bacteria killing capacity of plasma, our most functionally different measures (PHA response is an induced, mostly cellular, immune response while bactericidal capacity of plasma is constitutive and humoral), were significantly negatively correlated (*r* = −0.28) only in intensive farmlands in 2010, lending partial support to our prediction. This result points toward a trade-off between the innate and acquired immune system under certain conditions. This is different from the findings of Palacios et al. (2012), who only found evidence of a trade-off between cellular and humoral components of the innate immune system in tree swallows of Tompkins County, New York (USA). Nevertheless, other pair-wise correlations among immune markers were negatively correlated in high-quality habitat and uncorrelated in low-quality habitat, inconsistent with this hypothesis. Our results suggest that the answer may be more complex than a simple trade-off when resources are limited. Although trade-offs may be present when resources are limiting, negative correlations may also be caused by preferential investment in certain immune components appropriate for the pathogens present in the habitat. Further research is needed in order to determine the relative importance of resources and pathogens in a natural context.

Temporal environmental heterogeneity was also important. For example, the correlation between bacteria killing capacity and lymphocyte percentage was negative in 2010 and positive in 2011. Although 2 years of data are insufficient to clearly determine how the quality of each year influences the immune defenses, our results still suggest that immune markers might reveal opposing results even in the same population, the same habitat, but for different years. This is similar to the results of Hegemann et al. (2012) who found that patterns in immune functions of skylarks (*Alauda avensis*) differed between years.

At least two non-mutually exclusive explanations can account for the differences in relationship among immune measures across our farmland-year sub-groups. First, a difference in energetic or nutrient constraints could result from different habitat quality. Nutrition has been shown repeatedly to have an important impact on immune defenses (Lochmiller et al. 1993; Siva-Jothy and Thompson 2002). In our study system, Rioux Paquette et al. (In press) showed that insect prey abundance was lower late in the season in intensive farmlands when it is most crucial for nestling development and fledging. Yet this difference in prey abundances was only observed in 2 out of 3 years, likely as a result of yearly variations in meteorological conditions. Such differences in resources between years and habitats could explain the observed differences in the correlations among immune markers. Another possible explanation is that pathogen pressure differs among environments. In our system, the average number of Prot*ocalliphora* pupae per nest ranged from 9.7 ± 1.8 in intensive-2010 to 13 ± 2.6 in extensive-2011 with intermediate levels in intensive-2011 and extensive-2010 (6.3 ± 2.7 and 6.4 ± 2.2, respectively). However, the abundance of *Protocalliphora* pupae only differed between extensive-2010 and extensive-2011 (Tukey post-hoc test, *P* = 0.03). Parasites can have a considerable impact on immune measures (Boughton et al. 2011) and influence the correlation between the humoral and cellular immune responses in birds (Johnsen and Zuk 1999). For example, infection by coccidians, which are intracellular intestine parasites, significantly increased bacteria killing capacity, but not lysis or hemagglutination in house sparrows (Pap et al. 2011). Likewise, the presence of bacterial or viral pathogens is likely to influence investment in different axes of the immune system (Soler et al. 2011). Although only pathogen pressure from *Protocalliphora* (a large blood sucking ectoparasite) was measured in this study, the large variability in immune defense patterns observed is consistent with individuals modulating their immune components according to local environment in order to optimize their immune defenses.

### Integrated immune score and individual performance

Finding informative indicators of immunological health has been the holy grail of modern medicine and immunology for decades (Davis 2008). Although, this is fundamental both for medicine and eco-immunology, this is not a trivial task. Our article represents a modest attempt to do so by applying the novel approach proposed by Buehler et al. (2011). Correlations among immune measurements were inconsistent among farmland-year sub-groups. Using a single PCA of the pooled data for the whole population would thus be unjustified and could lead to erroneous conclusions (McCoy et al. 2006). Because PCAs were performed on each farmland-year sub-group to obtain an integrated immune score, none of the scores used as explanatory variables have the same meaning. Interpretation must then be made individually for each sub-group. While such an approach complicates the interpretation, it is more appropriate given that individuals from different sub-groups have probably experienced different ecological conditions (environment, pathogen pressure, nutritional status; which causes the differences in immune relationships).

Globally, relationships between the integrated immune score and performance were low. Variability in immune strategies within our farmland-year sub-groups could explain this lack of relationship, suggesting that adjustments within the immune system according to environmental conditions occurred on a very local scale. For example, the effect of the integrated immune score on fledging mass and growth was significant only in the intensive-2010 sub-group. It is difficult to conclude on the causes of this difference and it must be noted, however, that causation cannot be inferred from our analysis. Our results nevertheless suggest that the effect of immunity on performance could fluctuate with the environment. Consequently, it might be even more challenging to collapse information from different biomarkers into a metric that can be linked to fitness. Indeed, it is possible that individuals with large variation in their immune defense would perform better than others. Methods used to analyze complex systems may therefore offer an interesting way to push the envelope further in eco-immunology. For example, combining the concept of degeneracy (ability of different elements of a system to perform similar functions) and network theory has been proposed by Tieri et al. (2010) to explore the characteristics of such a complex system while taking into account the role of stochastic fluctuations.

Another factor which makes the study of immune defenses and individual performance difficult is mortality during early development. Most of the mortality occurs before all immune measures are obtained, and deceased individuals are thus not considered in analyses. Given that mean nestling mortality is relatively high in our study system and varies across habitats (intensive-2010: 28.0%; extensive-2010: 20.4%; intensive-2011: 37.3%; extensive-2011: 25.3%; see also Ghilain and Bélisle (2008)), measurement of immunity is partly based on a post-selection sample. Nestlings dying before fledging could have lower immune defenses (Christe et al. 1998). The non-random removal of individuals with the lowest integrated immune scores could explain the lack of correlation between our proxies of individual performance and immunity in intensive-2011, where mortality was highest. Even though we missed this invisible fraction (Nakagawa and Freckleton 2008) representing low fitness individuals, we were still able to detect a correlation between immunity and fledging mass or parasite burden in intensive-2010 and parasite burden in extensive-2011.

### Concluding remark

Our results suggest that eco-immunological studies on a particular species, or even a single population, will not be easily generalized to others. Given that spatiotemporal variation is likely to affect individual immune defenses, further studies on multiple immune measures are critically needed to quantify the importance of environmental variation on immune defenses. To understand the underlying mechanisms of organismal immunity, research must not only use multiple immune measurements, but also investigate how immune measures vary in different ecological contexts using larger scale, long-term studies in order to assess general trends. New statistical approaches might also be required to analyze data from multiple biomarkers. Molecular-based immune markers such as β-defensin genes (Hellgren and Sheldon 2011) or major histocompatibility complex diversity (Piertney and Oliver 2006) should also be included in studies to assess the complex interactions between genotype and environment as well as to tease apart the relative importance of evolutionary versus ecological variables involved in immune defense.

## Supporting Information

**Figure S1.** Redundancy analysis of pooled nestlings immune measurements with year, farm type and sex as constraining variables. Arrows represent loading values of the different immune measures. Red circles represent centroids of constraining variables. 20.37% of the variance is explained by RDA 1 and 2. *n* = 210.

**Figure S2.** Relationship between PC1 and mass at fledging of tree swallow nestlings. Regression lines are obtained from linear mixed model (LME) and represent the regression with a random intercept by nest-boxes. The contribution to PC1 of each immune marker by sub-group is equal to the horizontal loading displayed in Figure 2.

**Figure S3.** Relationship between PC1 and growth of tree swallow nestlings. Regression lines are obtained from linear mixed model (LME) and represent the regression with a random intercept by nest-boxes. The contribution to PC1 of each immune marker by sub-group is equal to the horizontal loading displayed in Figure 2.

**Figure S4.** Relationship between PC1 and parasite burden of tree swallow nestlings. Regression lines are obtained from linear model. The contribution to PC1 of each immune marker by sub-group is equal to the horizontal loading displayed in Figure 2. Gray area represents confidence interval.

**Table S1.** Eigenvalues of the redundancy analysis for the pooled value.

**Table S2.** Eigenvalues and loading values of the principal component analysis for the four sub-groups.
