# Supplementary material for: Ecological immunology in a fluctuating environment: an integrative analysis of tree swallow nestling immune defense
Source: Ecol Evol. 2013 Mar 9;3(4):1091–103. doi: 10.1002/ece3.504 (PMC3631416; doi:10.1002/ece3.504)
Supplement: Supplementary file 1 [file ece30003-1091-SD1.pdf]

Supplementary material

For

Ecological immunology in a fluctuating environment: an integrative analysis to tree swallow nestlings immune responses

Gabriel Pigeon, Marc Bélisle, Dany Garant, Alan A.Cohen and Fanie Pelletier

Table S1: Eigenvalues of the redundancy analysis for the pooled value.

	Eigenvalue	Proportion Explained
RDA1	0.755	0.151
RDA2	0.155	0.031
RDA3	0.022	0.004
PC1	1.124	0.225
PC2	0.918	0.184
PC3	0.874	0.175
PC4	0.646	0.129
PC5	0.507	0.507

Table S2: Eigenvalues and loading values of the principal component analysis for the four sub-groups.

Sub-group	Immune indices	Comp 1	Comp 2	Comp 3	Comp 4	Comp 5
Extensive 2010	Eigenvalue	1.296	1.123	1.046	0.826	0.71
	PHA	-0.033	0.713	0.483	0.445	-0.243
	B. killing	0.759	0.155	-0.144	0.314	0.529
	Hemolysis	0.598	0.453	-0.085	-0.600	-0.263
	Agglutination	-0.127	0.251	-0.879	0.288	-0.256
	% Lymphocyte	-0.587	0.567	-0.110	-0.293	0.485
Extensive 2011	Eigenvalue	1.747	1.104	0.879	0.798	0.472
	PHA	-0.059	0.793	0.551	-0.246	-0.057
	B. killing	0.756	0.181	-0.270	-0.377	0.425
	Hemolysis	0.359	-0.601	0.697	-0.074	0.136
	Agglutination	0.838	-0.047	-0.083	-0.168	-0.511
	% Lymphocyte	0.584	0.282	0.096	0.750	0.093
Intensive 2010	Eigenvalue	1.703	1.159	0.953	0.728	0.457
	PHA	-0.422	0.778	-0.193	-0.268	-0.328
	B. killing	0.832	-0.243	0.020	-0.044	-0.496
	Hemolysis	0.687	0.320	0.216	-0.548	0.282
	Agglutination	0.401	0.080	-0.897	0.091	0.143
	% Lymphocyte	-0.448	-0.621	-0.254	-0.588	-0.054
Intensive 2011	Eigenvalue	1.871	1.117	0.944	0.698	0.369
	PHA	-0.352	-0.420	0.792	0.258	-0.077
	B. killing	0.823	0.166	0.325	0.090	0.425
	Hemolysis	-0.360	0.680	0.427	-0.474	-0.025
	Agglutination	0.807	0.337	0.137	0.207	-0.416
	% Lymphocyte	0.537	-0.580	0.100	-0.597	-0.094

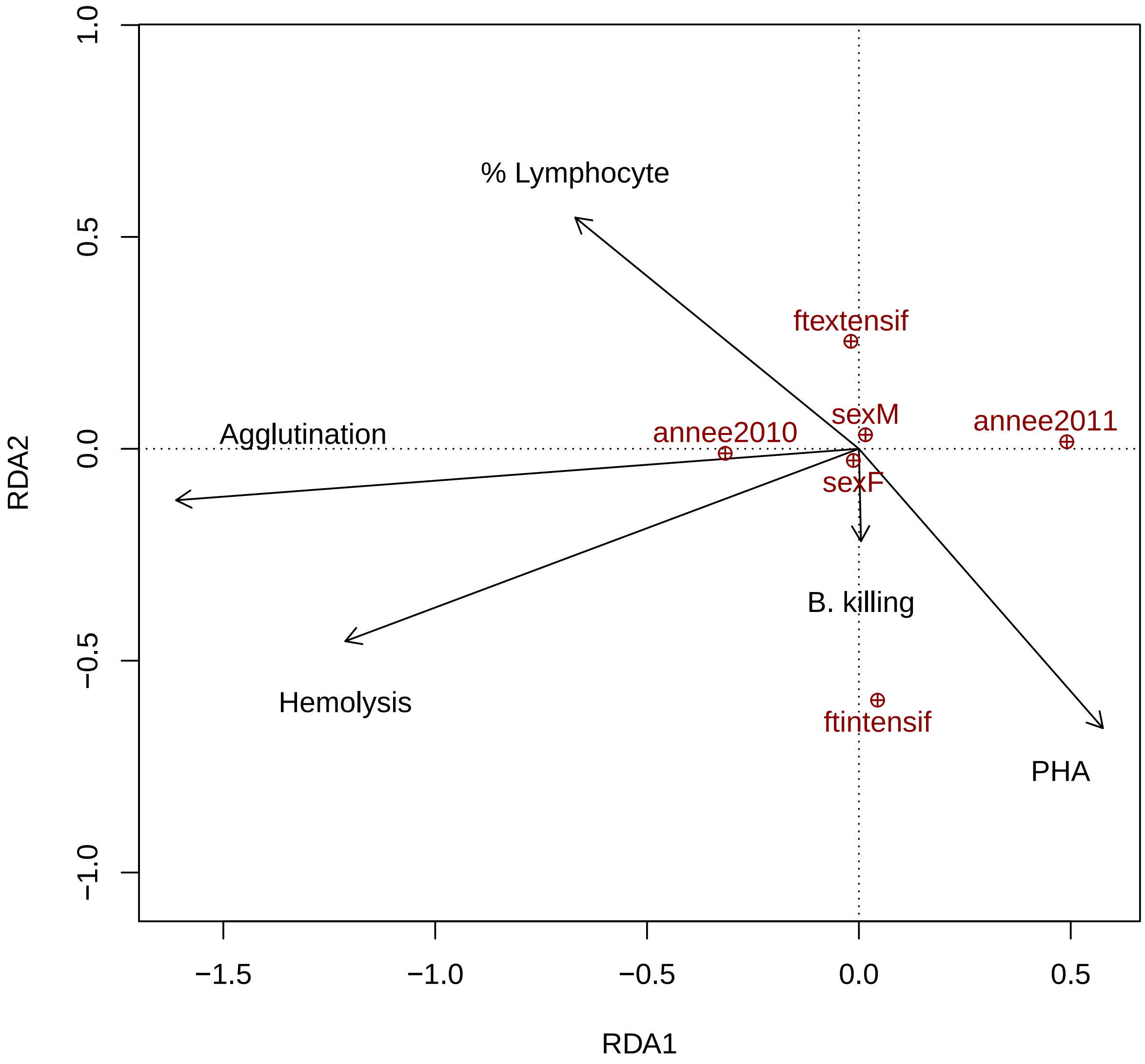

Figure S1: Redundancy analysis of pooled nestlings immune measurements with year, farm type and sex as constraining variables. Arrows represent loading values of the different immune measures. Red circles represent centroids of constraining variables. 20.37% of the variance is explained by RDA 1 and 2. n=210.

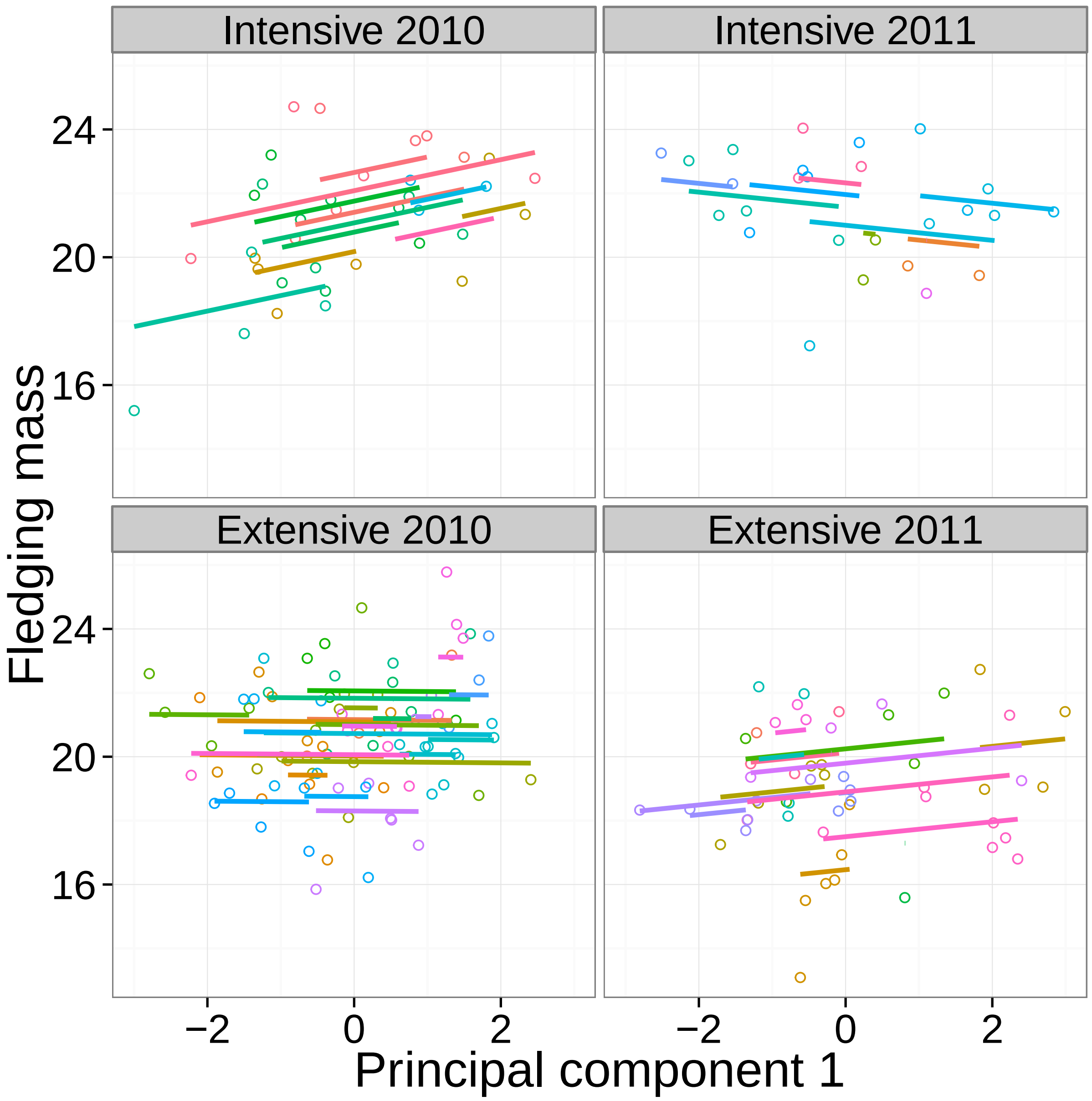

Figure S2: Relationship between PC1 and mass at fledging of tree swallow nestlings. Regression lines are obtained from linear mixed model (LME) and represent the regression with a random intercept by nest-boxes. The contribution to PC1 of each immune marker by sub-group is equal to the horizontal loading displayed in Figure 2.

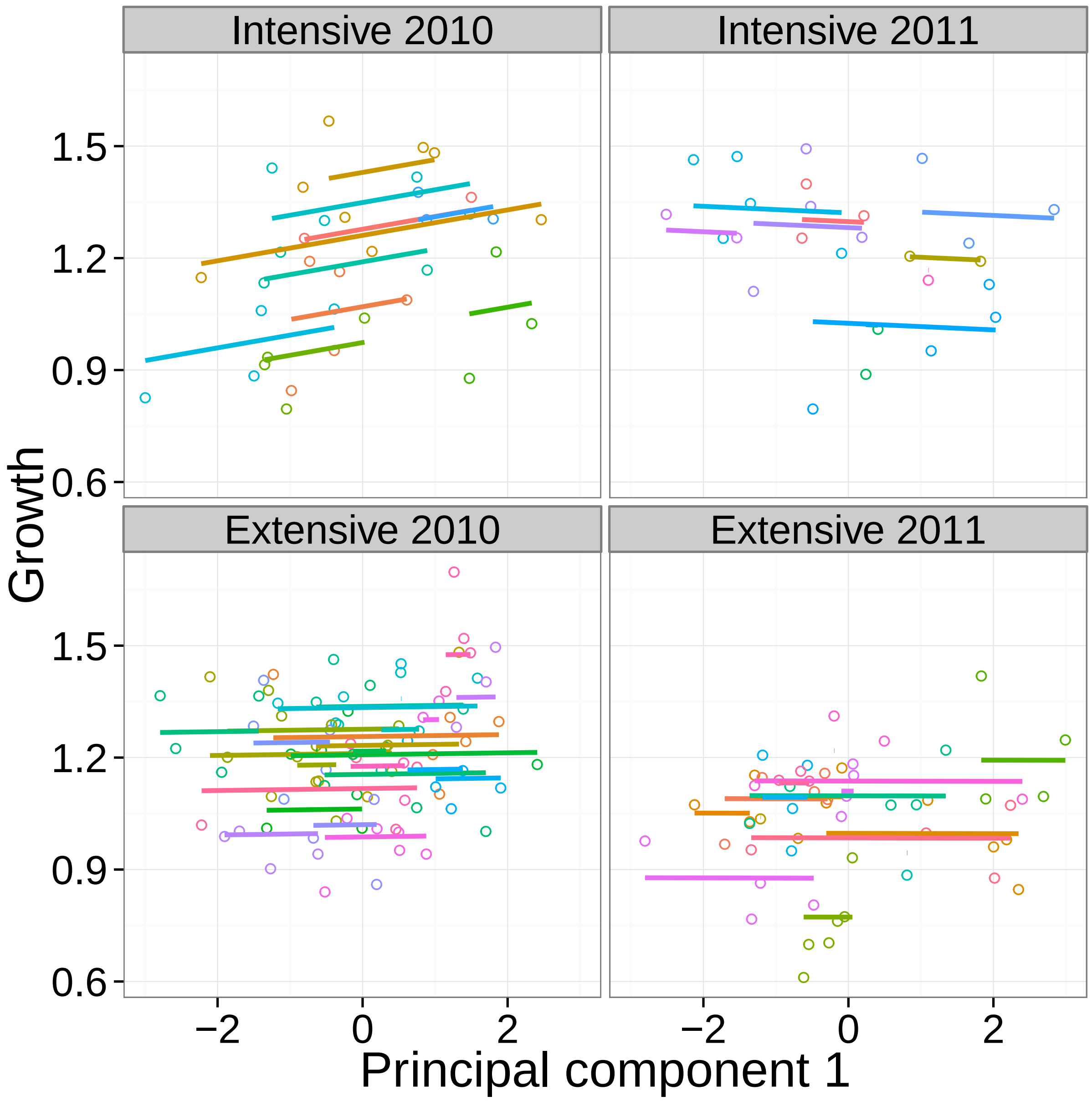

Figure S3: Relationship between PC1 and growth of tree swallow nestlings. Regression lines are obtained from linear mixed model (LME) and represent the regression with a random intercept by nest-boxes. The contribution to PC1 of each immune marker by sub-group is equal to the horizontal loading displayed in Figure 2.

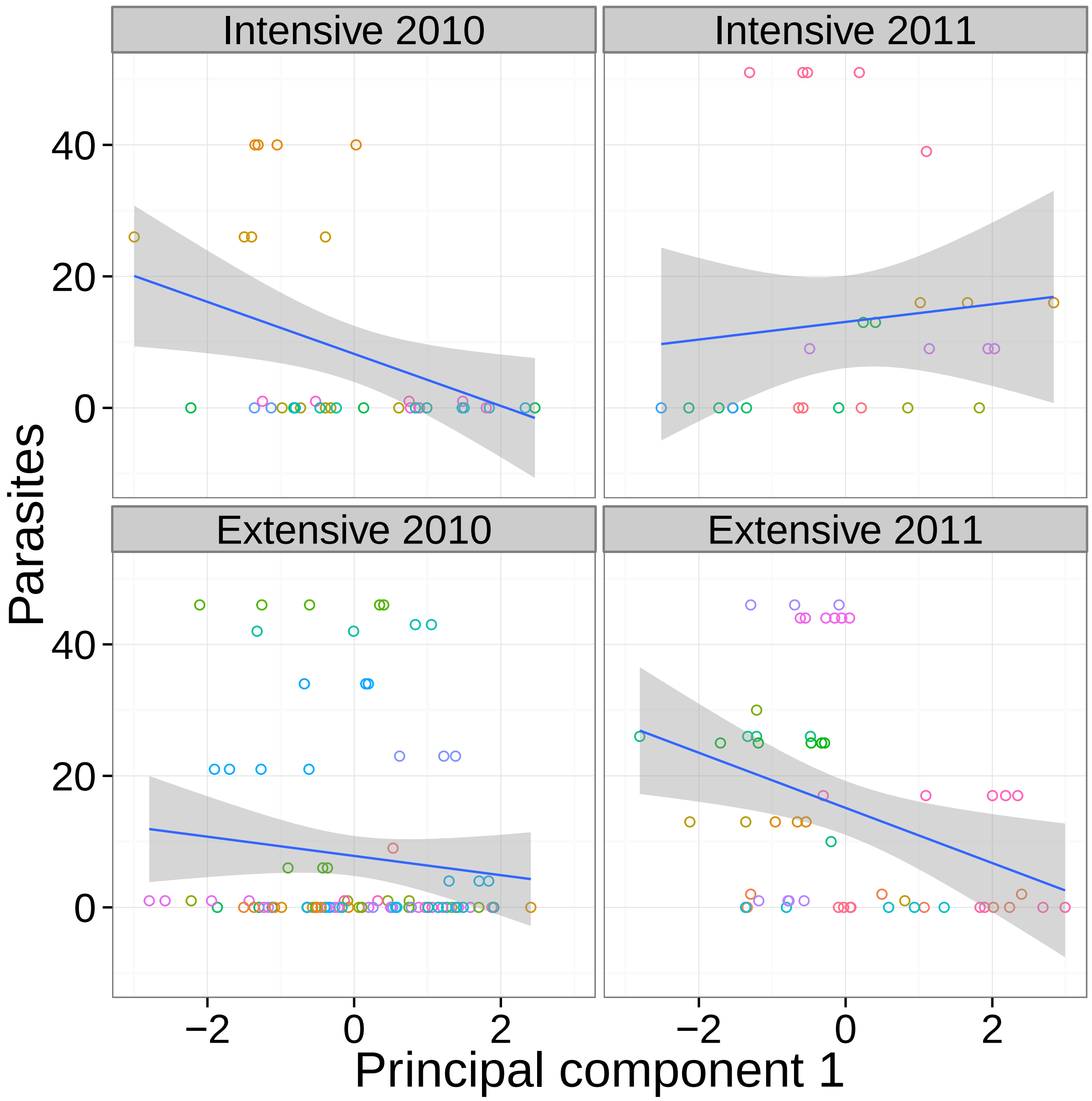

Figure S4: Relationship between PC1 and parasite burden of tree swallow nestlings. Regression lines are obtained from linear model. The contribution to PC1 of each immune marker by subgroup is equal to the horizontal loading displayed in Figure 2. Grey area represents confidence interval.
